# In silico mutational analysis to identify the role and pathogenicity of BCL-w missense variants

**DOI:** 10.1186/s43141-022-00389-2

**Published:** 2022-08-11

**Authors:** Poonam Kumari, Rashmi Rameshwari

**Affiliations:** Department of Biotechnology, Faculty of Engineering and Technology, Manav Rachna International Institute of Research and Studies, Faridabad, Haryana India

**Keywords:** Pro-survival, Pathogenicity, Missense variants, Destabilizing, Deleterious, Stability

## Abstract

**Background:**

Intrinsic pathway of apoptosis is generally mediated by BCL-2 (B cell lymphoma 2) family of proteins; they either induce or inhibit the apoptosis. Overexpression of BCL-2 in cancer cell may lead to delay in apoptosis. BCL-w is the pro-survival member of the BCL-2 family. BCL2L2 gene is present on chromosome number 14 in humans, and it encodes BCL-w protein; BCL-w protein is 193 amino acids residues in length. Interactions among the BCL-2 proteins are very specific. The fate of cell is determined by the ratio of pro-apoptotic proteins to pro-survival proteins. BCL-w promotes cell survival. Studies suggested that overexpression of BCL-w protein is associated with many cancers including DLBCL, BL, colorectal cancers, gastric cancers, and many more. The cause of overexpression is translocations or gene amplification which will subsequently result in cancerous activity.

**Process:**

For in-silico analysis, BCL2L2 gene was retrieved from UniProt (UniProt ID: Q92843). 54 missense variants have been collected in BCL-w proteins from COSMIC database. Different tools were used to detect the deleteriousness of the variants.

**Result:**

In silico mutational study reveals how the non-synonymous mutations directly affect the protein’s native structure and its function. Variant mutational analysis with PolyPhen-2 revealed that out of 55 variants, 28 of the missense mutations was probably damaging with a score ranging from 0.9 to 1, while 24 variants were benign with a score ranging from 0 to 0.4.

**Conclusions:**

This in silico work aims to determine how missense mutations in BCL-w protein affect the activity of the protein, the stability of the protein, and to determine the pathogenicity of the variants. Prediction of pathogenicity of variants will reveal if the missense mutation has a damaging effect on the native structure of protein or not. Prediction of protein stability will reveal whether the mutation has a stabilizing or destabilizing effect on the protein.

## Background

BCL-2 family of proteins are associated with mitochondrial-mediated cell death. The proteins of BCL-2 family either inhibits or induces cell death. On the basis of BH domain, members are classified into three groups [1]. The pro-survival proteins possess BH1-4 domains e.g. BCL-2, BCL-XL, MCL1 [2–4], BCL-w, and A1/BFL-1. Multi-domain pro-apoptotic proteins contains BH1-3 domains, e.g., BAX and BAK [2–5], and lastly the BH3 only pro-apoptotic proteins which are further classified as activators or sensitizers. BAD, BIK, BMF are sensitizers and BIM, tBID, and PUMA are activators [2, 6]. Here, sensitizers do not bind to BAK and BAX [2, 7, 8] while the BH3 domain of the activators binds to BAK and BAX and induces conformational change that results in the oligomerization of these proteins in the outer membrane of the mitochondria, this oligomerization results in MOMP formation [2, 9]. In cytosol, cytochrome c (released from mitochondria intermembraned space) with Apaf-1, caspase 9, and ATP [10–12] forms a complex also known as apoptosome. This complex cleaves off and activates the caspase 3 that results in apoptosis.

BCL-w is the pro-survival protein in the BCL-2 family. BCL2L2 gene present on chromosome number 14 in humans encodes the BCL-w protein and this protein is 193 amino acids residues in length [2, 13]. BCL-w protein is generally found on the outer membrane of the mitochondria [2, 14]. The BCL-w protein consists of nine α helices with flanking amphipathic helices α1 (10−24 residues), α2 (43−56), α3 (62−68), α4 (76−87), α6 (116−132), α7 (134−141), α8 (144−150), α9 (157−173), and central hydrophobic groove formed by helix, α5 (93−111).

BCL-w is found in the testes, colon, brains, and cells with lymphoid and myeloid origin [2, 13, 15]. Studies suggested that BCL-w is involved in spermatogenesis [2, 15] and is majorly expressed in spermatocytes, Leydig cells, Sertoli cells and spermatogonia, BCL-w also promotes their survival [2, 16, 17]. Experimental studies also suggest that overexpression of this protein might results in spermatocytes degeneracy, decline in the number of spermatogonia and vacuolization of sertoli cells [2, 18]. BCL-w also promotes the survival of gut epithelial cells [2, 15], prevents small intestine cells and mid-colon cells from death [2, 19], it also promotes enterocyte survival and B lymphocyte survival [2, 20]. High level of BCL-w also estimated in some areas of brain such as mature brain, sensory neurons, hippocampus and cerebellum [2, 21, 22]. BCL-w has also been involved in the development of dendrite and it controls the morphogenesis of mitochondria. BCL-w has also been involved in disorders of nervous system such as Alzheimer’s disease and Parkinson’s diseases, the cause of these diseases is the increased level of BCL-w. Overexpression of BCL-w is associated with ischemic brain [2, 23]. Overexpression of the BCL2L2 results in the survival of megakaryocytes and increased platelet formation [2, 24].

Genetic alterations in BCL2L2 contributes to many cancers such as copy number variations in small [2, 25] and non-small [2, 26] lung cancer, high level of BCL-w contributes to gastric carcinomas, and low BCL-w expression contributes to colorectal cancer [2, 27]. Patients with breast cancers significantly have high BCL-w mRNA level [2, 28, 29]. BCL-w has significantly involved with the cancer of urinary system [2, 30]. Overexpression of BCL-w is associated with cervical cancer, prostate cancer, hepatocellular carcinoma (HCC) and leiomyosarcomas. Expression of BCL-w is significantly higher in DLBCL, BL, CML [2, 31], and B-CLL [2, 32].

The interaction of pro-survival protein, i.e., BCL-w with pro-apoptotic proteins initiates the process of apoptosis but any dysregulation in these interactions will block the apoptotic pathway. Any chemical or amino acid alterations in the protein will interrupt the interactions between pro-survival proteins and pro-apoptotic proteins. Understanding of these mutations will help us to understand if the mutation is involved in any disease. This in silico study helps us to define the role of missense variants of BCL-w, which may alter proteins native structure and its function. By examining the role of mutation on biological function, we can determine the correlation between the mutation and the disease. The missense variants retrieved from this study were subjected to some in silico prediction tools such as Polyphen-2, SIFT, Provean, FATHMM, mutation assessor and stability prediction namely I-mutant 2.0, iStable, SAAFEC, SDM, DUET, and mCSM (Table 1).

**Table 1:** Stability predictions of missense variants using various prediction tools by using fasta format as input

S.No	Missense mutations	I-Mutant2.0	MUpro	SAAFEC	IStable
1	A159V	0.78 **Decrease**	− 0.383 **Decreasing**	− 0.04 **Destabilizing**	Increase
2	G154W	– 1.56 **Decrease**	− 0.332 **Decreasing**	− 0.38 **Destabilizing**	Increase
3	R161H	− 0.73 **Decrease**	− 1.345 **Decreasing**	− 0.80 **Destabilizing**	**Decrease**
4	E146K	− 1.11 **Decrease**	− 1.300 **Decreasing**	− 0.57 **Destabilizing**	**Decrease**
5	L180Q	− 2.55 **Decrease**	− 1.839 **Decreasing**	− 1.68 **Destabilizing**	**Decrease**
6	V178M	− 3.82 **Decrease**	− 0.277 **Decreasing**	− 0.83 **Destabilizing**	Increase
7	A177P	0.87 **Decrease**	− 1.188 **Decreasing**	− 0.95 **Destabilizing**	**Decrease**
8	S169P	0.36 Increase	− 1.818 **Decreasing**	− 0.02 **Destabilizing**	**Decrease**
9	A159P	− 0.61 **Decrease**	− 1.71 **Decreasing**	− 0.41 **Destabilizing**	**Decrease**
10	A7T	− 0.97 **Decrease**	− 0.700 **Decreasing**	− 0.65 **Destabilizing**	**Decrease**
11	A7G	− 0.98 **Decrease**	− 1.108 **Decreasing**	− 0.70 **Destabilizing**	**Decrease**
12	A7V	0.89 Increase	− 0.458 **Decreasing**	− 0.64 **Destabilizing**	Increase
13	P8L	0.57 Increase	− 0.546 **Decreasing**	− 0.54 **Destabilizing**	**Decrease**
14	A15T	− 1.37 **Decrease**	− 1.302 **Decreasing**	− 0.85 **Destabilizing**	**Decrease**
15	D16H	− 1.53 **Decrease**	− 2.114 **Decreasing**	− 0.22 **Destabilizing**	**Decrease**
16	R23K	− 1.38 **Decrease**	− 0.717 **Decreasing**	− 0.74 **Destabilizing**	**Decrease**
17	G34W	− 1.01 **Decrease**	0.524 Increase	− 0.70 **Destabilizing**	Increase
18	M46T	− 0.80 **Decrease**	− 1.556 **Decreasing**	− 2.46 **Destabilizing**	**Decrease**
19	M46I	0.25 Increase	− 0.826 **Decreasing**	− 1.11 **Destabilizing**	Increase
20	R47Q	− 0.07 **Decrease**	− 0.786 **Decreasing**	− 1.06 **Destabilizing**	Increase
21	G50R	− 0.24 **Decrease**	− 1.055 **Decreasing**	− 0.76 **Destabilizing**	Increase
22	G50V	− 0.00 Increase	− 1.074 **Decreasing**	− 1.05 **Destabilizing**	**Decrease**
23	E54K	− 1.96 **Decrease**	− 1.066 **Decreasing**	− 0.59 **Destabilizing**	**Decrease**
24	F57S	− 1.69 **Decrease**	− 2.031 **Decreasing**	− 2.68 **Destabilizing**	**Decrease**
25	R58Q	− 0.45 **Decrease**	− 0.878 **Decreasing**	− 0.71 **Destabilizing**	**Decrease**
26	R59C	− 0.29 **Decrease**	− 1.086 **Decreasing**	− 0.49 **Destabilizing**	**Decrease**
27	R59H	− 0.90 **Decrease**	− 1.488 **Decreasing**	− 0.58 **Destabilizing**	**Decrease**
28	S62F	0.30 Increase	− 0.682 **Decreasing**	− 0.36 **Destabilizing**	Increase
29	A66D	− 0.23 **Decrease**	− 0.766 **Decreasing**	− 0.66 **Destabilizing**	Increase
30	P72T	− 0.88 **Decrease**	− 0.976 **Decreasing**	− 1.10 **Destabilizing**	**Decrease**
31	S74L	2.04 Increase	0.475 Increasing	− 0.48 **Destabilizing**	Increase
32	Q76K	− 0.13 Increase	− 0.978 **Decreasing**	− 0.60 **Destabilizing**	**Decrease**
33	R78H	− 1.33 **Decrease**	− 0.917 **Decreasing**	− 0.86 **Destabilizing**	**Decrease**
34	S83F	1.35 Increase	− 0.158 **Decreasing**	− 0.67 **Destabilizing**	Increase
35	D84N	0.36 Increase	− 0.895 **Decreasing**	0.18 Stabilizing	Increase
36	N92Y	− 0.64 **Decrease**	0.137 Increasing	− 0.58 **Destabilizing**	Increase
37	R95S	− 1.76 **Decrease**	− 1.048 **Decreasing**	− 1.36 **Destabilizing**	**Decrease**
38	R95H	− 0.69 **Decrease**	− 1.092 **Decreasing**	− 1.11 **Destabilizing**	**Decrease**
39	S110R	− 0.39 Increase	− 0.722 **Decreasing**	− 0.80 **Destabilizing**	Increase
40	V111I	− 0.58 **Decrease**	− 0.480 **Decreasing**	− 0.33 **Destabilizing**	**Decrease**
41	V127M	− 1.16 **Decrease**	− 0.536 **Decreasing**	− 0.46 **Destabilizing**	**Decrease**
42	A128V	− 0.55 **Decrease**	− 0.296 **Decreasing**	0.09 Stabilizing	**Decrease**
43	E131G	− 0.84 **Decrease**	− 1.672 **Decreasing**	− 0.81 **Destabilizing**	**Decrease**
44	Q133R	− 0.06 **Decrease**	− 1.196 **Decreasing**	− 0.08 **Destabilizing**	Increase
45	A135V	− 0.77 **Decrease**	− 0.525 **Decreasing**	− 0.17 **Destabilizing**	Increase
46	S140C	− 0.23 Increase	− 0.575 **Decreasing**	− 0.26 **Destabilizing**	Increase
47	S141I	0.91 Increase	− 0.269 **Decreasing**	− 0.06 **Destabilizing**	Increase
48	G142E	− 1.09 **Decrease**	− 1.223 **Decreasing**	− 1.27 **Destabilizing**	**Decrease**
49	G152R	− 1.38 **Decrease**	− 0.671 **Decreasing**	− 0.94 **Destabilizing**	**Decrease**
50	R160W	− 0.67 **Decrease**	− 0.744 **Decreasing**	− 0.88 **Destabilizing**	**Decrease**
51	R161L	− 0.12 **Decrease**	− 0.316 **Decreasing**	− 0.44 **Destabilizing**	**Decrease**
52	R163W	− 0.55 **Decrease**	− 0.852 **Decreasing**	− 0.06 **Destabilizing**	**Decrease**
53	R171M	− 0.88 **Decrease**	− 0.328 **Decreasing**	− 0.44 **Destabilizing**	**Decrease**
54	V186A	− 3.07 **Decrease**	− 1.789 **Decreasing**	− 1.41 **Destabilizing**	**Decrease**
55	A188P	− 1.42 Increase	− 1.343 **Decreasing**	− 0.76 **Destabilizing**	Increase

Bold represents a destabilizing or decreased mutational effect by all the prediction tools used

## Method

### Data collection—selection of the BCL-w variants

For in silico analysis, BCL2L2 gene was retrieved from UniProt (UniProt ID: Q92843). 54 missense variants have been collected in BCL-w proteins from COSMIC database. Among these, neither of the variants were listed in the ClinVar.

### Variants pathogenicity prediction

For predicting the deleteriousness of the variants, the in silico pathogenicity prediction tools that were used were PolyPhen-2 [33], SIFT [34], Provean [35–37], FATHMM [38], and Mutation Assessor [39].

### Protein stability analysis

For predicting the of effect of amino acid change on the native BCL-w protein, I-mutant 2.0 [40], MUpro [41], and iStable [42], SAAFEC [43], SDM [44], DUET [45], and mCSM [46] web servers were used. I-mutant 2.0 is a web server that determines the change in stability due to point mutation or missense mutation. MUpro web server is a program that predicts the protein stability due to alteration in the sequence. Integrated predictor iStable was used for the predicting the stability of the protein, iStable may require both the sequence and the structure as an input. SAAFEC is a web server used to compute the energy changes due to single mutation. SDM (site-directed mutator) is an online server is that is also used for predicting the effect of point mutation on the protein stability. DUET is a web tool for the estimation of consequence of single mutation on proteins stability and its function. mCSM, a web tool used to estimate the impact of point mutation on protein stability, protein-protein-binding, and protein-DNA binding.

## Result

### Pathogenecity prediction of BCL-w missense variants

Variant mutational analysis with PolyPhen-2 revealed that out of 55 variants 28 of the missense mutations was probably damaging with score ranging from 0.9 to 1, while 24 variants were benign with score ranging from 0 to 0.4. PolyPhen-2 evaluates the damaging effect of point mutation by mapping SNPs to gene transcripts. From SIFT analysis, 28 out of 55 variants were deleterious, i.e., not tolerant with score ranging from 0 to 0.76, remaining 27 variants were tolerant (score range 0.76–1). Provean analysis revealed that 34 of the variants were neutral rest 20 were deleterious (one mutation, i.e., Q133R shows error) (Table 2). FATHMM analysis shows that 49 of the variants were deleterious, i.e., with score ≥ 0.67 rest 6 variants were neutral, i.e., no impact on the proteins native structure and function. Mutation assessor tool predicts the impact of point mutation on protein sequence and has revealed that 29 variants have low value while 15 variants have medium effect and 11 mutations have neutral effect.

**Table 2:** Computational pathogenicity prediction scores of BCL-w variants

S.No	Position	PolyPhen-2	SIFT	Provean	Fathmm	Mutation assessor
1	A159V	0.659 Probably damaging	1.00 Tolerant	− 1.031 Neutral	1.06	1.39 Low
2	G154W	0.938 Probably damaging	0.50 Not Tolerant	− 2.206 Neutral	0.90	1.39 Low
3	R161H	0.993 Probably damaging	1.00 Tolerant	− 1.065 Neutral	0.91	1.1 Low
4	E146K	0.365 Benign	0.94 Tolerant	− 0.118 Neutral	1.06	0.69 Neutral
5	L180Q	1.00 Probably damaging	0.94 Not tolerant	− 2.021 Neutral	0.70	1.67 Low
6	V178M	0.014 Benign	1.00 Not Tolerant	− 0.512 Neutral	0.78	1.5 Low
7	A177P	0.996 Probably damaging	1.00 Tolerant	− 1.640 Neutral	0.90	1.735 Low
8	S169P	0.998 Probably damaging	1.00 Tolerant	− 1.302 Neutral	0.97	1.735 Low
9	A159P	0.973 Probably damaging	1.00 Tolerant	− 1.477 Neutral	0.97	1.39 Low
10	A7T	0.001 Benign	0.38 Tolerant	0.093 Neutral	0.98	− 0.205 Neutral
11	A7G	0.003 Benign	0.38 Tolerant	− 0.272 Neutral	0.95	0.345 Neutral
12	A7V	0.018 Benign	0.38 Tolerant	− 1.56 Neutral	1.04	0 Neutral
13	P8L	0.028 Benign	0.38 Tolerant	− 0.548 Neutral	1.09	0.755 Neutral
14	A15T	0.519 Possibly damaging	0.94 Tolerant	− 0.676 Neutral	1.02	1.78 Low
15	D16H	0.965 Probably damaging	0.94 Not tolerant	− 2.623 Deleterious	0.64	1.905 Low
16	R23K	0.012 Benign	0.88 Tolerant	0.024 Neutral	0.91	0.205 Neutral
17	G34W	0.999 Probably damaging	1.00 Not tolerant	− 2.283 Neutral	0.78	0.825 Low
18	M46T	0.997 Probably damaging	1.00 Not tolerant	− 3.453 Deleterious	0.88	2.215 Medium
19	M46I	0.360 Benign	1.00 Not tolerant	− 0.984 Neutral	1.07	1.87 Low
20	R47Q	0.562 Possibly damaging	1.00 Tolerant	− 2.933 Deleterious	0.76	1.56 Low
21	G50R	1.000 Probably damaging	1.00 Not Tolerant	− 6.246 Deleterious	0.51	2.88 Medium
22	G50V	1.000 Probably damaging	1.00 Not tolerant	− 6.669 Deleterious	0.60	2.185 Medium
23	E54K	1.000 Probably damaging	1.00 Tolerant	− 2.725 Deleterious	0.99	2.855 Medium
24	F57S	0.964 Probably damaging	1.00 Not tolerant	− 5.229 Deleterious	0.97	2.215 Medium
25	R58Q	0.138 Benign	1.00 Tolerant	− 1.238 Neutral	0.93	2.215 Medium
26	R59C	0.001 Benign	1.00 Not tolerant	− 5.428 Deleterious	1.09	0.645 Neutral
27	R59H	0.099 Benign	1.00 Not tolerant	− 2.921 Deleterious	1.12	1.65 Low
28	S62F	0.993 Probably damaging	1.00 Not tolerant	− 4.105 Deleterious	0.86	2.25 Medium
29	A66D	0.001 Benign	1.00 Tolerant	− 1.132 Neutral	1.09	1.055 Low
30	P72T	0.986 Probably damaging	1.00 Not tolerant	− 6.239 Deleterious	0.87	2.805 Medium
31	S74L	0.557 Probably damaging	1.00 Tolerant	− 2.282 Neutral	0.81	1.795 Low
32	Q76K	0.142 Benign	1.00 Tolerant	− 1.504 Neutral	1.00	1.395 Low
33	R78H	0.280 Benign	1.00 Tolerant	− 2.066 Neutral	1.20	2.125 Medium
34	S83F	0.001 Benign	1.00 Not tolerant	− 0.852 Neutral	1.19	1.39 Low
35	D84N	0.073 Benign	1.00 Tolerant	− 1.032 Neutral	1.15	1.48 Low
36	N92Y	1.000 Probably damaging	1.00 Not tolerant	− 7.001 Deleterious	0.43	2.925 Medium
37	R95S	0.994 Probably damaging	1.00 Not tolerant	− 5.221 Deleterious	0.14	2.965 Medium
38	R95H	0.997 Probably damaging	1.00 Not tolerant	− 4.147 Deleterious	0.15	2.275 Medium
39	S110R	1.000 Probably damaging	1.00 Not tolerant	− 3.767 Deleterious	1.18	2.545 Medium
40	V111I	0.254 Benign	1.00 Tolerant	− 0.981 Neutral	0.84	1.795 Low
41	V127M	0.985 Probably damaging	1.00 Not tolerant	− 1.422 Neutral	0.84	1.745 Low
42	A128V	0.000 Benign	1.00 Tolerant	− 0.721 Neutral	0.96	0.435 Neutral
43	E131G	0.034 Benign	1.00 Tolerant	− 2.283 Neutral	1.08	1.645 Low
44	Q133R	0.000 Benign	1.00 Tolerant	Error	1.10	0.11 Neutral
45	A135V	0.067 Benign	1.00 Tolerant	− 1.765 Neutral	1.13	1.5 Low
46	S140C	0.987 Probably damaging	1.00 Not tolerant	− 3.590 Deleterious	0.87	2.16 Medium
47	S141I	0.000 Benign	1.00 Not tolerant	− 3.534 Deleterious	0.95	1.245 Low
48	G142E	0.996 Probably damaging	1.00 Not tolerant	− 6.273 Deleterious	− 1.69	2.875 Medium
49	G152R	0.999 Probably damaging	0.94 Not tolerant	− 3.741 Deleterious	0.85	1.445 Low
50	R160W	1.000 Probably damaging	1.00 Not tolerant	− 3.749 Deleterious	0.91	1.355 Low
51	R161L	0.945 Possibly damaging	1.00 Tolerant	− 2.168 Neutral	1.00	1.1 Low
52	R163W	1.000 Probably damaging	1.00 Not tolerant	− 1.314 Neutral	0.94	0.69 Neutral
53	R171M	0.406 Benign	0.75 Not tolerant	− 1.019 Neutral	0.97	0.69 Neutral
54	V186A	0.972 Probably damaging	0.62 Not tolerant	− 1.041 Neutral	0.03	1.39 Low
55	A188P	0.264 Benign	0.62 Tolerant	− 1.173 Neutral	0.01	1.795 Low

Note: PolyPhen-v2 score less than 0.5 is considered to be tolerated and more than 0.5 is considered to be deleterious. SIFT score ranges from 0.0 to 0.05 are considered to be deleterious while score near 1.0 are considered to be tolerated; Provean score equals to or below − 2.5 are considered to be deleterious while score above − 2.5 are considered to be neutral; FATHMM score equals to or above 0.67 are deleterious; mutation assessor score prediction: 0–1 is neutral, 1–2 low, and above 2 medium.

### Protein stability analysis

Pathogenic missense mutations cause change in free energy which further leads to alteration in protein stability. Here, BCL-w variants were subjected to various protein stability tools for analyzing change in free energy due to point mutation. I-Mutant 2.0, MUpro, iStable, SAAFEC, SDM, DUET, and mCSM tools were used for determining the protein stability. The tools revealed that the variants decrease the protein stability by showing a destabilizing or decreasing energy as result. I-Mutant2.0, MUpro, mCSM, SDM, DUET, and SAAFEC tools shows the more negative ΔΔG value (ΔΔG > 0) shows the more destabilizing effect of the mutation, while the more positive ΔΔG value (ΔΔG <0) shows stability decrease in case of iStable tool.

**Fig. 1:**
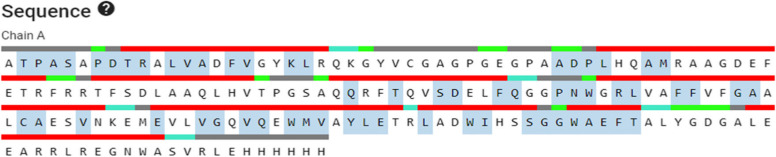
The amino acid sequence of BCL-w protein retrieved from RCSB PDB databank

Some of the servers require fasta format while some require PDB structure or PDB ID as an input. I-Mutant 2.0, MUpro, iStable, and SAAFEC use fasta format while SDM, DUET, and mCSM need PDB structure or PDB ID as an input. Some post-translational modifications that takes place during the conversion of peptide sequence to 3D structure may cause deletion of amino acids residue, i.e., some part of the protein may not be included in the crystallographic structure, as small peptide sequence yields a better crystal quality or structure of a protein is extracted from a crystal structure from proteins complex and isolating some proteins from complex of proteins may cause differences in the sequence in fasta format to sequence in PDB structure. Now, the fasta format of BCL-w starts from MATPA, while amino acid sequence in PDB structure starts from ATP, as shown in Fig. 1 for this reason, mutation given in DUET, SDM, and mCSM as A158V instead of A159V, besides this some of the amino acids are not included in the sequence of PDB structure due to these modifications are Q132R, V185A, and A187P as shown in Table 3.

**Table 3:** DUET, mCSM, and SDM stability scores of BCL-w variants by using PDB format as input

S.No	Variants	SDM (ΔΔG value in Kcal/mol)	DUET (ΔΔG value in Kcal/mol)	mCSM (ΔΔG value in Kcal/mol)
1	A158V	− 0.24 **Destabilizing**	0.108 Stabilizing	− 0.245 **Destabilizing**
2	G153W	− 0.28 **Destabilizing**	− 1.013 **Destabilizing**	− 1.167 **Destabilizing**
3	R160H	0.05 Stabilizing	− 1.148 **Destabilizing**	− 1.305 **Destabilizing**
4	E145K	− 0.46 **Destabilizing**	− 0.072 **Destabilizing**	− 0.372 **Destabilizing**
8	S168P	0.09 Stabilizing	− 0.073 **Destabilizing**	− 0.247 **Destabilizing**
9	A158P	− 3.0 **Destabilizing**	− 0.587 **Destabilizing**	− 0.245 **Destabilizing**
10	A6T	− 0.31 **Destabilizing**	− 0.333 **Destabilizing**	− 0.623 **Destabilizing**
11	A6G	− 0.24 **Destabilizing**	− 0.121 **Destabilizing**	− 0.385 **Destabilizing**
12	A6V	− 0.21 **Destabilizing**	− 0.255 **Destabilizing**	− 0.519 **Destabilizing**
13	P7L	− 0.32 **Destabilizing**	− 0.043 **Destabilizing**	− 0.308 **Destabilizing**
14	A14T	− 1.97 **Destabilizing**	− 0.734 **Destabilizing**	− 0.735 **Destabilizing**
15	D15H	0.35 Stabilizing	− 0.281 **Destabilizing**	− 0.546 **Destabilizing**
16	R22K	− 0.26 **Destabilizing**	− 0.78 **Destabilizing**	− 1.064 **Destabilizing**
17	G33W	0.04 Stabilizing	− 0.977 **Destabilizing**	− 1.242 **Destabilizing**
18	M45T	− 1.8 **Destabilizing**	− 1.22 **Destabilizing**	− 1.375 **Destabilizing**
19	M45I	− 0.03 **Destabilizing**	− 0.273 **Destabilizing**	− 0.784 **Destabilizing**
20	R46Q	− 0.17 **Destabilizing**	− 0.262 **Destabilizing**	− 0.522 **Destabilizing**
21	G49R	− 0.76 **Destabilizing**	− 0.694 **Destabilizing**	− 0.91 **Destabilizing**
22	G49V	0.47 Stabilizing	1.008 Stabilizing	0.49 Stabilizing
23	E53K	− 0.46 **Destabilizing**	− 0.166 **Destabilizing**	− 0.46 **Destabilizing**
24	F56S	− 3.23 **Destabilizing**	− 2.492 **Destabilizing**	− 2.231 **Destabilizing**
25	R57Q	− 0.44 **Destabilizing**	0.044 Stabilizing	− 0.059 **Destabilizing**
26	R58C	− 0.27 **Destabilizing**	− 0.319 **Destabilizing**	− 0.239 **Destabilizing**
27	R58H	0.29 Stabilizing	− 0.778 **Destabilizing**	− 0.833 **Destabilizing**
28	S61F	0.8 Stabilizing	− 0.651 **Destabilizing**	− 1.042 **Destabilizing**
29	A65D	− 0.94 **Destabilizing**	− 1.004 **Destabilizing**	− 1.205 **Destabilizing**
30	P71T	− 0.38 **Destabilizing**	− 0.346 **Destabilizing**	− 0.623 **Destabilizing**
31	S73L	1.24 Stabilizing	0.383 Stabilizing	− 0.146 **Destabilizing**
32	Q75K	0.17 Stabilizing	0.431 Stabilizing	− 0.054 **Destabilizing**
33	R77H	− 0.22 **Destabilizing**	− 1.464 **Destabilizing**	− 1.529 **Destabilizing**
34	S82F	0.64 **Destabilizing**	− 0.253 **Destabilizing**	− 0.543 **Destabilizing**
35	D83N	0.31 Stabilizing	− 0.637 **Destabilizing**	− 0.989 **Destabilizing**
36	N91Y	0.35 Stabilizing	− 0.403 **Destabilizing**	− 0.546 **Destabilizing**
37	R94S	− 3.2 **Destabilizing**	− 2.82 **Destabilizing**	− 2.249 **Destabilizing**
38	R94H	− 0.82 **Destabilizing**	− 2.229 **Destabilizing**	− 2.091 **Destabilizing**
39	S109R	0.1 Stabilizing	− 0.313 **Destabilizing**	− 0.78 **Destabilizing**
40	V110I	0.36 Stabilizing	− 0.313 **Destabilizing**	− 0.78 **Destabilizing**
41	V126M	− 0.11 **Destabilizing**	− 0.015 **Destabilizing**	− 0.239 **Destabilizing**
42	A127V	− 1.03 **Destabilizing**	− 0.25 **Destabilizing**	− 0.395 **Destabilizing**
43	E130G	− 1.53 **Destabilizing**	− 0.956 **Destabilizing**	− 0.802 **Destabilizing**
44	Q132R	–	–	–
45	A134V	− 0.97 **Destabilizing**	− 0.359 **Destabilizing**	− 0.51 **Destabilizing**
46	S139C	0.71 Stabilizing	0.227 Stabilizing	− 0.225 **Destabilizing**
47	S140I	2.13 Stabilizing	0.382 Stabilizing	− 0.467 **Destabilizing**
48	G141E	− 2.58 **Destabilizing**	− 0.705 **Destabilizing**	− 0.463 **Destabilizing**
49	G151R	0.14 Stabilizing	− 0.177 **Destabilizing**	− 0.607 **Destabilizing**
50	R159W	0.59 Stabilizing	− 0.531 **Destabilizing**	− 0.736 **Destabilizing**
51	R160L	− 0.08 **Destabilizing**	0.142 Stabilizing	− 0.022 **Destabilizing**
52	R162W	0.63 Stabilizing	− 0.757 **Destabilizing**	− 1.082 **Destabilizing**
53	R170M	0.14 Stabilizing	− 0.186 **Destabilizing**	− 0.073 **Destabilizing**
54	V185A	–	–	–
55	A187P	–	–	–

Bold represents destabilizing or decreased effect of the mutation

## Discussion

Present in silico mutational study reveals how the non-synonymous mutations directly affect the proteins native structure and its function. The activity of the protein complex and its function depends on the complex formed between proteins; the interactions between proteins might be necessary for molecular features like cell signaling and cell regulation. The protein complex formed may be homodimer or heterodimer are formed due to interactions between proteins. The missense mutations at the interface of the protein-protein interaction (PPI) causes disruption in the shape, size, and secondary structure of the complex. For the specific function of the protein complex, there should be presence of stable interaction between proteins. Moreover, mutation of large amino acids into a smaller amino acid causes gaps while mutation of smaller one leads to bumps or inter-molecular clashes. BCL-w, has a pro-survival function, and is also involved in normal as well as diseased cells and disorders of nervous system and cancer. The protein–protein interactions gets disturbed due to non-synonymous mutation which may lead to diseased state. The structure of the protein is directly influenced by its function and its stability. The genetic variations, i.e., amino acid change that represses its property directly influences all other properties. The hydrogen bonds within amino acid residues maintains the protein stability, i.e., reduced H-bonds may cause loss of stability of the protein while higher H-bonds may increase the protein stability. The structural changes caused due to variants corresponds to physicochemical properties of the proteins like size, charge, hydrophobicity, molecular weight, and side chains. These changes further causes alteration in the chemical properties which may be necessary for maintaining secondary, tertiary, and quaternary structure of proteins.

Most pathogenic variants destabilizes the 3D structure, stability, and folding-free energy of the protein, which subsequently results in disruption in proteins function and regulation [47, 48].

## Conclusion

Proteins are dynamic in nature as they are flexible in nature due to temperature, pH, and interaction with other molecule may be a ligand. Understanding of proteins native conformation may reveal the role of variants in diseased condition. The activity and function of the protein complex is determined by its interaction with other proteins. However, the stability of a protein complex can disrupt due to mutations in the protein. This in silico study has estimates the efficiency of various pathogenicity prediction tools and stability analysis tools for BCL-w variants and the study may help in characterization of mutations in the protein complex and molecular level. Furthermore, the result indicates that the missense mutation alters the stability of BCL-w.

## Data Availability

NA

## Abbreviations

MCL-1: Myeloid cell leukemia-1
BCL-w: B cell lymphoma-w
A1/BFL-1: BCL-2-related protein A1/BCL-2 related isolated from fetal liver-11
BAX: BCL-2-associated X protein
BAK: BCL-2 antagonist/killer
BAD: BCL-2-associated agonist of cell death
BIK: BCL-2 interacting killer
BMF: BCL-2-modifying factor
BIM: BCL-2-interacting mediator of cell death
tBID: Truncated form of BH3-interacting domain death agonist
PUMA: p53-upregulated modulator of apoptosis
MOMP: Mitochondrial outer membrane permeabilization
CML: Chronic myeloid lymphoma
Apaf-1: Apoptosis protease activating factor 1
B-CLL: B cell chronic lymphocytic leukemia
PolyPhen-2: Polymorphism phenotyping-2
SIFT: Sorting intolerant from tolerant
Provean: Protein variation effect analyzer
FATHMM: Functional analysis through hidden Markov models
